# Impact psychologique des femmes enceintes atteintes du COVID-19: à propos de 2 cas cliniques

**DOI:** 10.11604/pamj.2021.39.271.30846

**Published:** 2021-08-25

**Authors:** Cyrine Belghith, Souhir Najar, Raouia Haouel, Sawssam Armi, Sihem Bouzidi, Nahla Ben Saada, Leila Attia, Tahar Makhlouf, Olfa Slimani, Nabil Mathlouthi

**Affiliations:** 1 Service de Gynécologie Obstétrique A, Hôpital Charles Nicolle, Tunis, Tunisie,; 2 Faculté de Médecine de Tunis, Tunis, Tunisie,; 3 Cellule d'Assistance Psychologique, Hôpital Charles Nicolle, Tunis, Tunisie

**Keywords:** Grossesse, COVID-19, psychologie, confinement, cas clinique, Pregnancy, COVID-19, psychology, confinement, clinical case

## Abstract

Ce travail de recherche est une lecture psychologique d'inspiration psychanalytique de deux cas de femmes enceintes atteintes du COVID-19 à des termes de grossesses différents appartenant au service de gynécologie-obstétrique A, hôpital Charles Nicolle. Notre étude a été réalisée entre l'année 2020 et 2021, jusqu'à l'accouchement des deux femmes. Deux jeunes femmes Tunisiennes âgées de 28 et 30 ans ont été déclarées COVID positive au cours de leurs grossesses. Elles ont subi un choc émotionnel. Dans ce travail, nous avons abordé différents points de collusion entre la vie et la mort en exposant des présentations détaillées du vécu de ces deux femmes durant leur confinement.

## Abstract (en)

This study is based on a psychoanalytically inspired psychological investigation of two pregnant women with COVID-19 at different stages of pregnancy in the Department of Obstetrics and Gynaecology A at the Charles Nicolle Hospital. Our study was conducted between 2020 and 2021, until deliveries. Two young Tunisian women aged 28 and 30 years were tested positive for COVID-19 during pregnancy. They suffered from emotional shock. In this study, we discuss the different points of collision between life and death by describing, in detail, the experiences of these two women during their confinement.

## Introduction

Le coronavirus SARS-CoV-2 mis en évidence en fin d´année 2019 en Chine a atteint tous les continents. En janvier 2020, l´Organisation mondiale de la santé (OMS) a déclaré que l´épidémie du COVID-19 était une urgence de santé publique internationale avec un fort risque de dissémination dans de nombreux pays à travers le monde et de ce fait, l´a requalifiée en pandémie le 11 mars 2020 [1]. L´infection par le virus de la COVID-19 est le plus souvent à l´origine d´un syndrome infectieux bénin, associant à différents degrés des symptômes (fièvre, toux, myalgies, céphalées et éventuels troubles digestifs), voire totalement asymptomatique. Cependant le SARS-CoV-2 peut être à l´origine de pathologies pulmonaires graves et parfois de décès. Ceci a entrainé la saturation des institutions hospitalières et une augmentation dramatique de la mortalité au sein même des services de soins.

## Patient et observation

### Observation Nº1

**Information spécifique à la patiente**: notre patiente était âgée de 29 ans, femme au foyer, niveau socio-économique moyen, mariée depuis 16 mois. GIIPI, une grossesse arrêtée révisée. Elle était enceinte à 32 semaines d´aménorrhée. 40 Jours après une grossesse arrêtée à 8 semaines d´aménorrhée, vécue avec beaucoup de peine et d´anxiété, notre patiente se déclarait être enceinte à nouveau. C´était une grossesse non programmée. Au cours de son contrôle échographique de routine, un raccourcissement du col était diagnostiqué. Un repos strict était indiqué. La patiente a été hospitalisée dans notre service pour menace d´accouchement prématuré sévère. Etant donné son état de santé limitant ses mouvements, elle était à chaque fois dépendante des aides-soignantes et des ouvrières pour subvenir à ses besoins. La durée de la grossesse n´était pas paisible. La patiente a vécu plusieurs imprévues comme une menace d´avortement d´où l´indication d´un cerclage à chaux en urgence à 20 semaines d´aménorrhée et un diabète gestationnel. Elle a vécu sa grossesse avec le risque éminent de perdre son bébé tant désiré. Elle avait peur de revivre le même traumatisme de sa fausse couche précédente. La grossesse de notre patiente était caractérisée par des plaintes somatiques et le besoin excessif de réaliser des échographies et des enregistrements du rythme cardiaque fœtal. Une surveillance rapprochée et stricte était instaurée jusqu´à 36 semaines d´aménorrhée. Devant l´amélioration clinique et la maturité fœtale, la patiente était mise sortante avec programmation de son accouchement à 39 semaines d´aménorrhée.

**Résultats cliniques**: une semaine plus tard, la patiente se présentait aux urgences accompagnée par son mari en état de détresse aigu rapportant qu´elle avait les symptômes du COVID-19 et qu´elle avait peur de perdre son bébé à cause du virus. La patiente affichait une grande agitation psychomotrice, elle était agressive et accusait le service de l´avoir contaminée, ceci était rapporté avec beaucoup de larmes et de cris. Sur le plan clinique, la patiente présentait une dyspnée, des courbatures et une toux sèche. Elle était persuadée d´avoir attrapé le COVID-19.

**Évaluation diagnostique**: la patiente était placée dans le secteur COVID en attendant son résultat de réaction en chaîne par polymérase (PCR). Elle interagissait avec le monde externe à travers une fenêtre. Dès que le résultat de son PCR était confirmé positif, Elle développait une anxiété avec des signes somatiques: une détresse respiratoire, suivie par des pleurs.

**Intervention thérapeutique**: toute au long de son confinement, la patiente a été suivie par la psychologue de la cellule d´assistance psychologique par le biais d´une téléconsultation. La durée de la séance était entre 30 et 45 minutes et la fréquence des appels pouvaient aller jusqu'à 4 fois par semaine. Isolée dans le circuit COVID, la patiente avait besoin de tisser un lien avec le monde externe. Ses craintes tournaient autour de son bébé et le risque de le contaminer. Elle exprimait une grande colère à l´égard du service de ne pas avoir assuré sa sécurité alors qu´elle était un cas fragile. Elle faisait souvent des recherches par rapport à la transmission du virus de la mère au bébé. Elle culpabilisait de l´avoir mis en danger. La patiente développait un trouble du sommeil et une perte d´appétit lié à son anosmie et agueusie (des symptômes qui se sont manifestés à long terme).

**Suivi et résultats**: à 39 Semaines d´aménorrhée, la patiente a accouché par césarienne d´un nouveau-né en bonne santé prénommée « Ghalia » (précieuse). La patiente a rapporté que la santé de sa fille sera toujours sa préoccupation primaire à cause du COVID-19.

### Observation Nº2

**Information spécifique à la patiente**: la patiente était âgée de 30 ans, personnel de la santé, mère d´un enfant âgé de 5ans. Elle était enceinte à 22 semaines d´aménorrhée. La patiente travaillait au sein d´un service qui est passé par une période pandémique, où presque une vingtaine du personnel de santé ont été contaminés. Durant cette période, notre patiente était affectée aux urgences du service, elle était enceinte à 22 semaines d´aménorrhée. Etant donné qu'elle était en contact direct avec une personne testée positive, elle a réalisé un test PCR. Elle était asymptomatique.

**Résultats cliniques**: deux jours après, elle était déclarée positive. À l´annonce de son résultat, elle s´est complètement effondrée, elle était submergée par la terreur, ses pensées étaient confuses et a toute de suite pensé à sa petite famille et spécialement son petit garçon. L´état de santé du bébé n'était pas évoqué. Elle s'est confinée chez elle. Elle était isolée seule dans une chambre de peur de contaminer son fils et son mari. C'est son conjoint qui lui ramenait à manger. Leur interaction était séparée par une porte.

**Evaluation diagnostique**: la patiente n´a pas présenté des symptômes organiques alarmants. Elle était plus impactée sur le plan psychologique. Ce vécu a été ramené avec beaucoup d'émotion.

**Intervention thérapeutique**: la soignante a été prise en charge sur le plan psychologique toujours par le biais d´une téléconsultation. La durée de la séance était de 45 minutes, et à une fréquence de 2 fois par semaine. La patiente souffrait de la séparation brutale avec son fils. Tout au long de son confinement, elle a présenté plusieurs répercussions psychologiques: culpabilité, angoisse de mort, sensation d´étouffement, insomnie, et perte d´appétit. La future maman, a fini par réclamer l´arrêt de la grossesse, sous prétexte qu'il ne sera jamais un bébé en bonne santé à cause du COVID-19 et que sa situation va être plus complexe en étant enceinte. Toutes les préoccupations rapportées par la future mère tournaient autour de son enfant, ses questionnements sur la situation étrange, son incompréhension sur l'état de santé de sa mère et la raison pour laquelle il ne peut pas l´approcher et l´embrasser. Il vivait la situation comme un abandon à cause du futur bébé.

**Suivi et résultats**: elle a accouché sans incidents, c´était un bébé en bonne santé. La patiente décrivait son bébé comme « un bébé COVID ».

## Discussion

**Grossesse, COVID-19 et statistique**: les maladies émergentes telles que la COVID-19, sont de nos jours encore plus au cœur de l´actualité et constituent une incitation permanente à mieux connaître et mieux gérer les émergences virales. Elles suscitent la crainte des populations mais demeurent un véritable défi pour le corps scientifique mondial. En effet, les effets de la pandémie de COVID-19 sur la santé mentale ne sont pas encore entièrement connus.

Une revue récente de la littérature a montré chez les patients infectés par le COVID-19, des symptômes de stress post-traumatique (92,2% chez 714 patients hospitalisés et stables), et un taux plus élevé de symptômes dépressifs (29,2% chez 57 patients récemment guéris, contre 9,8% chez 50 individus en quarantaine) et d'anxiété (26,7%). Les patients ayant des antécédents psychiatriques ont présenté une réactivation des symptômes [1]. Des recherches sur cet impact sur la population et ses différentes sections sont nécessaires. Il est important d´identifier les groupes les plus touchés et les méthodes les plus efficaces dans la prévention et le traitement des troubles mentaux en cas de pandémie [2].

**Maladie, traumatisme et maternité**: en analysant ces deux cas cliniques dans un contexte du COVID-19, nous avons observé que l´annonce d´un résultat positif à ce virus était accueillie par un choc émotionnel au début, suivi par une colère intense exprimée par le besoin de désigner à chaque fois un coupable. Le personnel de santé du service pour la première patiente et le temps passé aux urgences selon la deuxième patiente. En effet, ceci pourrait être interprété par la nouvelle perception du mot positif, un mot qui porte en lui l'idée de la mort réelle [3, 4].

**De l´angoisse de mort à la culpabilité maternelle**: en raison de l´absence des données scientifiques, on se trouve face à l´ambigüité de l´impact du virus COVID-19 sur la grossesse d´où les interrogations des patientes au sujet du risque de transmission, et des complications materno-fœtales. La culpabilité est le sentiment en commun entre les deux cas présentés. Une culpabilité liée à l´éventuelle transmission de ce virus à son bébé et de le mettre en danger. Les deux patientes ont manifesté un état anxieux qui se caractérise par les symptômes suivants: a) une gêne thoracique et une sensation d'étouffement; b) une insomnie; c) une agitation psychomotrice; d) une irritabilité; e) un état d´hypovigilance; f) un sentiment d´insécurité permanent lié à des pensées négatives envahissantes; g) les plaintes somatiques comme des douleurs au ventre (halluciner des contractions). Un tiraillement entre la perte d'appétit liée à l´agueusie ou aux éléments dépressifs et la peur d'entrainer une dénutrition au bébé.

Ceci a été accentué par le risque de l´accouchement prématuré annoncé par les médecins traitant. Ces constatations ont été confirmées par les résultats d´une revue de la littérature faite par Julia *et al*. qui indiquent une prévalence significative de la dépression (56,3%) et les symptômes d'anxiété (77%) chez les femmes enceintes pendant la pandémie de COVID-19 [5].

**Impacte de la collision COVID-19/grossesse sur l´interaction mère-bébé**: on ne peut nier le fait que le profil psychologique de la femme enceinte est d'emblée très particulier. Bydlowsky et Golse qualifient ce phénomène par la « transparence psychique » [6]. Le fonctionnement psychique de la femme enceinte est «marqué par un surinvestissement de son histoire personnelle et de ses conflits infantiles». En effet, la grossesse réactive chez la future mère « le Moi-bébé »c´est à dire l´enfant qu´elle était, et fait émerger des représentations préconscientes et inconscientes au tour de son bébé à naitre.

Toutefois, la maladie vient faire éruption dans la psyché de la mère. Ceci se traduit dans le premier cas par une angoisse de perte réactivée à chaque fois par non seulement les problématiques obstétricales rencontrées au cours de sa grossesse mais aussi la contamination par le virus. Sachant qu'elle souffre encore d´un état de stress post-traumatique lié à sa grossesse arrêtée auparavant. La patiente portait une grossesse précieuse avec beaucoup de symbolique fantasmatique ébranlé par le COVID-19. Quant au deuxième cas, elle a présenté une ambivalence par rapport au désir de sa grossesse, ceci se traduit par le désir d´avorter et son inquiétude sur l´état de santé du bébé à long terme. Enfin, les restrictions sanitaires imposées à la patiente, comme l´abstention à toucher son fils est vécu avec une grande souffrance. Elle dégage des projections, face au comportement décrit de son enfant « il est très attaché à moi, on se n´est jamais séparé ». C'est peut-être elle qui a du mal à s´en détacher. Heureusement, que la séparation était temporaire et le deuil était vite résolu. Alors que l'isolement du premier cas était vécu comme une contenance affective et sécuritaire pour elle et son bébé.

**Informations pour les femmes enceintes et les futures mères**: les informations destinées aux femmes enceintes et aux nouvelles mères doivent aborder les questions suivantes: a) incertitude sur COVID-19 et sur l´impact qu´il peut avoir sur la grossesse et la santé périnatale. Ces informations doivent provenir de sources faisant autorité et être fondées sur des preuves [7]. b) Recommandations concernant les comportements d´isolement social, l´allaitement et les impacts des catastrophes sur la santé mentale et physique. c) La façon dont les soins médicaux et les pratiques de sages-femmes sont fournis à l'époque de COVID-19 et les méthodes d'effectuer des visites (au cabinet et en ligne). d) Des informations sur la manière d´obtenir de l´aide et/ou un soutien psychologique [8]. e) Procédures de prévention des infections pour le travail, l´accouchement et le post-partum. L´information doit être fournie le plus tôt possible: avoir accès à l´information peut alléger le stress et l´anxiété subséquente [9]. f) Informations sur la santé mentale et les moyens de soulager le stress chez les femmes enceintes et encourager l´utilisation de sources fiables [10].

Différentes manières de fournir des informations devraient être utilisées, y compris les technologies de l´information (sites Web du gouvernement, ministère de la santé, établissements de santé), des lignes d´assistance téléphonique gratuites répondant aux questions des femmes enceintes et les informations fournies par les professionnels de santé lors des visites tant au cabinet en ligne, ainsi que lors de la prise de rendez-vous. En effet, la téléconsultation a permis de maintenir le contact avec les femmes désireuses d´obtenir une grossesse, de la poursuivre ou non, et d´assurer leur surveillance et leur prise en charge, malgré les distances. Par ailleurs, il a été démontré que les femmes qui avaient plus de connaissances sur COVID-19 fournies par des professionnels de la santé ont montré des niveaux inférieurs d'anxiété et de symptômes dépressifs [11].

Etant donné que les femmes enceintes étaient très tôt considérées comme une population à risque face au COVID-19, le taux d´anxiété et de dépression observés chez ces femmes a beaucoup augmenté partout dans le monde. Vient s´ajouter à cela l´histoire de la grossesse et l'accouchement qui peuvent être considérés comme des événements potentiellement stressants, voire traumatisants, nécessitant que les femmes doivent faire face aux changements de leurs modes de vie, de leurs habitudes et même de leurs images de soi et identité.

## Conclusion

L´épidémie due au coronavirus, qui a contraint la population au confinement pendant deux mois, lors de la 1^re^ et 2^e^ vague, a eu des conséquences psychologiques sur toute la population, notamment sur les femmes enceintes. Les caractéristiques de cette pandémie (rapidité de diffusion, connaissances incertaines, sévérité, morts de soignants) ont installé un climat anxiogène. Des facteurs organisationnels peuvent être source de stress: déficit d´équipement de protection individuel, réaffectation de postes, manque de communication, pénurie de matériels de soins, bouleversement de la vie quotidienne familiale et sociale. D´autres facteurs de risque sont identifiés comme l´absence de soutien, la crainte de contaminer un proche, l´isolement ou la stigmatisation sociale et le haut niveau de stress au travail. Devenir mère tout en ayant le COVID-19 constitue une menace pour la qualité du lien mère-bébé. Etre déclaré positif en étant enceinte, le danger se fait doublement sentir parce qu´il y a un risque de perte réelle, c'est ainsi que cette maladie prend la valeur de la représentation de la séparation. Ceci nous amène à poser l´hypothèse de la place de ce virus dans ce lien. Les parturientes ont ainsi un risque augmenté d´anxiété, de dépression, d´épuisement, d´addiction et de trouble de stress post-traumatique.

## References

[1] Chan-Chee Ch, Léon Ch, Lasbeur L, Lecrique J, Raude J, Arwidson P (2020). La santé mentale des français face au COVID-19: prévalences, évolutions et déterminants de l´anxiété au cours des deux premières semaines de confinement. Front Psychol.

[2] Hotopf M, Bullmore E, O´Connor RC, Holmes EA (2020). The scope of mental health research during the COVID-19 pandemic and its aftermath. Br J Psychiatry.

[3] Molgora S, Fenaroli V, Prino L E, Rollé L, Sechi C, Trovato A (2018). Fear of childbirth in primiparous Italian pregnant women: the role of anxiety, depression, and couple adjustment. Women Birth.

[4] Bendrihen N (2011). En cancérologie: l´épreuve du réel. Le Psychologue en Service De Médecine.

[5] Julia S, Maria N, Paweł B, Dorota Ł, Katarzyna W, Sara S (2021). Perinatal mental health during COVID-19 Pandemic: an Integrative review and implications for clinical practice. J Clin Med.

[6] Bydlowski M, Golse B (2001). De la transparence psychique à la préoccupation maternelle primaire : une voie de l´objectalisation. Le carnet Psy.

[7] Liu X, Chen M, Wang Y, Sun L, Zhang J, Shi Y (2020). Prenatal anxiety and obstetric decisions among pregnant women in Wuhan and Chongqing during the COVID-19 outbreak: a cross-sectional study. Bjog Int J Obstet Gynaecol.

[8] Farewell CV, Jewell J, Walls J, Leiferman JA (2020). A mixed-methods pilot study of perinatal risk and resilience during COVID-19. J Prim Care Community Health.

[9] Preis H, Mahaffey B, Heiselman C, Lobel M (2020). Pandemic-related pregnancy stress and anxiety among women pregnant during the coronavirus disease 2019 pandemic. Am J Obstet Gynecol MFM.

[10] Berthelot N, Lemieux R, Garon-Bissonnette J, Drouin-Maziade C, Martel É Maziade M (2020). Uptrend in distress and psychiatric symptomatology in pregnant women during the coronavirus disease 2019 pandemic. Acta Obstet Gynecol Scand.

[11] Kahyaoglu Sut H, Kucukkaya B (2021). Anxiety, depression, and related factors in pregnant women during the COVID-19 pandemic in Turkey: a web-based cross-sectional study. Perspect Psychiatr Care.

